# Developing an mHealth Application to Coordinate Nurse-Provided Respite Care Services for Families Coping With Palliative-Stage Cancer: Protocol for a User-Centered Design Study

**DOI:** 10.2196/34652

**Published:** 2021-12-13

**Authors:** Aimee R Castro, Antonia Arnaert, Karyn Moffatt, John Kildea, Vasiliki Bitzas, Argerie Tsimicalis

**Affiliations:** 1 Ingram School of Nursing McGill University Montreal, QC Canada; 2 School of Information Studies McGill University Montreal, QC Canada; 3 Gerald Bronfman Department of Oncology Faculty of Medicine and Health Sciences McGill University Montreal, QC Canada

**Keywords:** respite care, caregivers, cancer, neoplasms, user-centered design, mobile applications, palliative care, home care services, information systems research framework, hospice and palliative care nursing

## Abstract

**Background:**

Patients living with palliative-stage cancer frequently require intensive care from their family caregivers. Without adequate community support services, patients are at risk of receiving inadequate care, and family caregivers are at risk for depression and poor health. For such families, in-home respite care can be invaluable, particularly when the services are flexible and staffed by trusted care providers, such as nurses. Other industries are using mobile apps to make services more flexible. However, few apps have been developed to coordinate nurse-provided respite care services, and to our knowledge, none have been designed in conjunction with families affected by cancer.

**Objective:**

The aim of this study is to develop a mobile health (mHealth) app prototype for coordinating flexible and trusted in-home respite care services provided by nurses to families coping with palliative-stage cancer in Québec, Canada.

**Methods:**

This user-centered design research comprises the core component of the *iRespite Services iRépit* research program. For this study, we are recruiting 20 nurses, 15 adults with palliative-stage cancer, and 20 of their family caregivers, from two palliative oncology hospital departments and one palliative home-care community partner. Overseen by an Expert Council, remote data collection will occur over three research phases guided by the iterative Information Systems Research Framework: Phase 1, brainstorming potential app solutions to challenging respite care scenarios, for better supporting the respite needs of both family caregivers and care recipients; Phase 2, evaluating low-fidelity proofs of concept for potential app designs; and Phase 3, usability testing of a high-fidelity interactive proof of concept that will then be programmed into an app prototype. Qualitative and quantitative data will be descriptively analyzed within each phase and triangulated to refine the app features.

**Results:**

We anticipate that preliminary results will be available by Spring 2022.

**Conclusions:**

An app prototype will be developed that has sufficient complimentary evidence to support future pilot testing in the community. Such an app could improve the delivery of community respite care services provided to families with palliative-stage cancer in Québec, supporting death at home, which is where most patients and their families wish to be.

**International Registered Report Identifier (IRRID):**

PRR1-10.2196/34652

## Introduction

### Background

Cancer is the leading cause of death in both Canada and Québec, with nearly 50% of Canadians developing the disease at some point in their lives [1,2]. Cancer symptoms often result in patients relying heavily on the skilled assistance of their family caregivers to continue living in the community, where most palliative care patients want to be [3-5]. However, without adequate support services, patients are at higher risk of receiving inadequate care and for costly hospital readmissions if their care becomes impossible to manage at home [6,7]. Simultaneously, family caregivers encounter a high risk of negative role consequences, including sleep deprivation, depression, reduced immunity, and early-onset mortality [6,8,9]. These risks are heightened during the palliative stage of cancer, when management of complex symptoms is prioritized over curative treatments [6,9].

As the number of cancer cases in Québec continues to rise [2], in-home respite care can be a crucial support service for families [7,10]. Respite care services offer opportunities for caregivers and care recipients to experience short breaks from each other and their caregiving/care-receiving family roles, while another person provides care [11,12]. Yet, based on our preliminary research, including literature reviews and discussions with directors of palliative and respite care organizations, the current landscape of these services in Québec is fragmented, with services often being difficult to access [5]. Most families accessing respite care services pay out-of-pocket, creating a potential affordability barrier [13,14]. Furthermore, respite care services often have inflexible hours, and they are typically staffed by home care providers who lack clinical expertise [10,15-17]. As a result of these barriers, respite care services are often underused, especially by families managing complex medical cases such as palliative-stage cancer [12,15].

Families coping with palliative-stage cancer require easily scheduled respite care services staffed by trusted providers [15,16,18]. Nursing is consistently ranked as the most respected and trusted profession by the public [19]. With their extensive clinical and theoretical training, nurses may be best positioned to provide trusted respite care services to families coping with complex care conditions [16,18]. Furthermore, these nursing services could be flexibly scheduled with opportunities to personalize the services received, by mobilizing the capabilities of mobile health (mHealth) apps [20,21].

This context warrants the creation of a new mHealth app to optimize the flexible coordination of respite care services in Québec, beginning with nurse-provided services for palliative-stage cancer. Other service providers, such as Airbnb and DoorDash, are using apps to improve service coordination by facilitating communication and scheduling. However, we have not identified any apps in academia or industry that focus on providing respite care services to families coping with cancer. Moreover, we have only identified one app in the research literature for specifically coordinating nurse-provided respite care services for families affected by age-related chronic conditions [21]. Therefore, the aim of this study is to develop an mHealth app prototype for coordinating flexible and trusted in-home respite care services, provided by nurses to families coping with palliative-stage cancer in Québec. This study has been awarded a Rossy Cancer Network Care, Quality, and Innovation research fund grant (2020) to support the work described (Multimedia Appendix 1).

### Study Design and Framework

**Figure 1:**
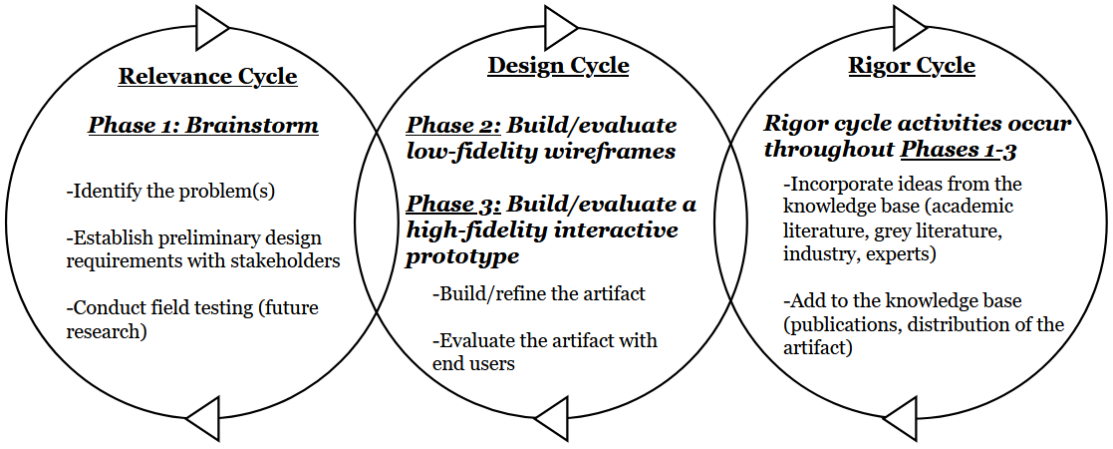
An adaptation of the Information Systems Research Framework [26], with its three methodological cycles, which will guide the proposed study.

Following ethical approval, a user-centered design study will be conducted over three phases to develop a rigorous and relevant app prototype [22-25]. An Expert Council composed of the research team and five key informants will oversee the study. Phase 1 will consist of brainstorming how an app might be used to address families’ needs, given various respite care scenarios. Phase 2 will involve wireframing of several low-fidelity proof-of-concept app designs and prioritizing key features. Phase 3 will consist of designing and testing the usability of a high-fidelity interactive proof of concept (ie, the online design will be “clickable”), which will then be programmed into a functional app prototype. The cyclical Information Systems Research Framework [26] has been adapted to inform each study phase (Figure 1). The iterative and integrative cycles of this framework consist of the (1) relevance cycle**,** composed of research activities supporting end-user app refinement; (2) rigor cycle, where external knowledge and research is synthesized to inform the app design; and (3) design cycle**,** where the app is built into a functional prototype.

### Objectives

The objectives are as follows: (1) to explore participants’ perspectives on the relevance of mHealth for the provision of nurse-provided respite care services, and (2) to design a rigorous and relevant proof of concept of a mHealth app for coordinating trusted and flexible respite care services, provided by nurses, to families coping with palliative-stage cancer, and (3) to conduct usability testing on the interactive proof of concept to support the development of a functional app prototype.

### Description of the Potential App

The development of this app comprises the core component of the *iRespite Services iRépit* research program led by the manuscript authors. Depending on participants’ needs identified throughout the study, the resulting app prototype could facilitate advanced and flexible scheduling for respite care with the same nurse-providers, or perhaps even offer on-demand scheduling. We predict that the final prototype will include features to support separate but integrated processes (ie, dashboards) focused on the needs of the two primary end users of the app: family caregivers and nurses. The dashboard for family caregivers will likely allow caregivers to sign up and directly schedule nurse-provided respite care services, with opportunities to request a nurse with specific skills (eg, experience caring for patients with a specific type of cancer) or payment option (eg, nurses whose services may be reimbursed through insurance). The dashboard for nurses will likely allow the nurses to sign up, describe their skills and certifications, and indicate their availabilities to provide respite care. However, since this study will incorporate ongoing end-user participation, we anticipate that our current predictions of the prototype features will differ significantly from the final prototype design.

## Methods

### Sampling Methods

#### Target Sampling Networks

The targeted web-based sampling networks will comprise the patient, family, and nursing networks of two palliative oncology hospital departments and one palliative home-care community partner in Montreal, Québec.

#### Participant Eligibility

The recruited sample will be composed of (1) family caregivers of adults living with palliative-stage cancer (“family caregivers”), (2) adults living with palliative-stage cancer (“care recipients”), (3) registered nurses (“nurses”), and (4) key informants.

Inclusion criteria for all participants will consist of adults (aged 18 years or older) who live in Québec. *Family caregivers* must self-identify as a family caregiver providing in-person care to a person diagnosed with cancer who is receiving palliative care services or is known to the palliative care teams of the target sampling networks. Family caregivers may also be up to 6 months post bereavement for a person diagnosed with cancer who had received palliative care services via the target sampling networks. *Care recipients* will be cancer patients who have a family caregiver providing them with regular in-person care. Care recipients will be either receiving palliative care services or known to the palliative care teams of the target sampling networks. *Registered nurses* will consist of nurses who are licensed in Québec and who have experience in providing home care, palliative care, respite care, and/or oncology care. *Key informants* will be identified by the research team as having relevant knowledge and expertise related to the management and deployment of the overall project.

Exclusion criteria for all participants will be that they (1) are not comfortable speaking and reading in English or French, (2) are unable to provide consent, or (3) do not have access to an internet-connected device capable of videoconferencing.

#### Sample Size

##### Sample Sizes for Phases 1 and 2 Focus Groups and Interviews

The participant numbers and research activities for each research phase are displayed in Multimedia Appendix 2.

A total of 30 participants (10 nurses, 10 family caregivers, and 10 care recipients) will be needed for the Phase 1 focus groups and individual interviews. These same participants will be invited to participate in Phase 2 focus groups and interviews. Focus groups for formative user-centered design research should be large enough to encourage brainstorming among diverse, representative target end-users, but these groups should be no larger than 12 participants [23,27]. Therefore, a total of 10 participants for each major type of focus group (nurse, family caregiver, and care recipient), further divided into English or French focus groups, should offer appropriate focus group sizes for the proposed research.

##### Sample Sizes for Phase 3 Usability Testing

Phase 1 and 2 nurses and family caregivers will be invited to participate in Phase 3. An additional 5 nurses and 5 family caregivers will be recruited for individual usability testing to provide new perspectives on the interactive proof of concept [23,25], for a total of 15 nurses and 15 family caregivers participating in this phase. A sample size of 15 in each sample subgroup is estimated to identify at least 90% of usability problems in artifact design [28,29].

##### Expected Recruitment for This Study, Accounting for Attrition Rates

Attrition rates for palliative care studies that are conducted over the course of several months to over 1 year can range from 24% [30] to 63% [31]. Flexible research strategies, videoconferencing, and in-home data collection can increase enrolment and reduce attrition in the palliative care population [32-34]. Our research will be implementing these strategies of virtual and in-home data collection, which should improve participant enrolment and retention in our study.

We anticipate that family caregivers will have similar retention rates to those of care recipients, given how intertwined family caregiver and care recipient roles are [32]. Assuming a 50% attrition rate for each group of participants over the course of the study, we expect to recruit 15 care recipients, 20 family caregivers, and 20 nurses in total, to achieve the above sample sizes for each phase. With 5 key informants recruited for the Expert Council, the total sample size will be 60 participants recruited remotely across the study sites.

#### Recruitment

In the current context of COVID-19, this study has been adapted to recruit and collect data solely on the web. Purposive sampling will be used to recruit potential participants via the targeted nursing-, respite-, and cancer-related networks [23,35]. Collaborators within these target networks will be requested to share the bilingual study brief with nurse employees in the networks, as well as with families receiving palliative care services, via the associated social networks and institutional apps of the target networks (ie, the organizational social media accounts; email listservs; workplace communications; intranets; institutional apps; and on-site television screens). The study brief will contain bilingual links to the study Qualtrics contact forms for interested family caregivers, care recipients, and nurses to follow up with the team. Key informants will be directly recruited via email by the doctoral candidate on this study.

Eligible recruits who follow up using the Qualtrics contact form, as well as key informants who indicate interest, will be contacted by a member of the research team to set up a videoconferencing appointment to further explain the study. Once they have received information about the study purpose and scope, informed consent will be sought by all participants through a Qualtrics e-consent form.

Participants will be purposively chosen based on a few demographic questions that will be included in the consent forms. Family caregivers and care recipients will be chosen to achieve sample diversity according to age [36], cancer typology [37], and gender [36,38]. These factors are known to affect individuals’ cancer caregiving and care receiving experiences [36-38], as well as their perspectives on mHealth supports [39,40]. Nurses will be purposively recruited to ensure a diversity of relevant perspectives on palliative care, oncology nursing, respite care, and home care services [41,42].

Purposively chosen participants will be contacted by email, a mutually agreed upon focus group or individual interview date will be arranged, and a videoconferencing invitation will be sent. Once the target size and diversity of the sample have been achieved, any additional eligible recruits will be placed on a waitlist for future inclusion, should participant attrition of the original 30 participants from Phase 1 occur.

Each participant will be offered a CAD $25 (US $19.55) gift card for either Visa or Mastercard following each interview or focus group that they choose to participate in [43]. A CAD $500 (US $391) stipend will be offered to each key informant at the end of the study, following their participation in the fourth Expert Council meeting and their ongoing advisement on the study. Key informants will be asked to provide a maximum of 15 hours of work over the course of the study [43].

### Data Collection

#### Setting

Participant data will be collected remotely using videoconferencing software. All Expert Council meetings, focus groups, individual interviews, and usability test sessions will be video-recorded using Microsoft Teams or Zoom built-in recording functionalities, to record participant interactions with the different app designs. Although we will encourage key informants and participants to keep their video cameras on, they will be allowed to turn off their video cameras if they choose to do so. All meetings will also be audio-recorded for backup using a voice recorder. Focus groups, individual interviews, and usability testing sessions of the proof of concept should last between 60 to 90 minutes. The interviewer (Phases 1 and 2) or test session guide (Phase 3) will be PhD candidate ARC and/or a member of the research team. Another member of the research team will record field notes during data collection, recording observations about what participants see, say, and do [23,44].

#### Phases 1 to 3: Rigor Cycle 1 (Ongoing)

Literature and app store reviews are presently ongoing and will continue throughout the three phases with the support of a librarian scientist. Google Scholar and Google Search Engine alerts have been set up to receive notifications of new, relevant data sources to further inform the design of the proofs of concept and the development of the functional app prototype.

#### Phase 1: Brainstorm mHealth Solutions to Respite Care Scenarios

##### Relevance Cycle 1: Determine Respite Care Problem Scenarios and Brainstorm Together

During the first Expert Council meeting, the key informants will review the study materials prior to the recruitment of other participants. The review of the study materials by the key informants will help ensure that the proposed study is designed to meet the needs of end users and other stakeholders. In this first meeting, the Expert Council will also determine 2 to 3 brief respite care video scenarios to be created using animation software, such as Doodly [45]. These videos will be discussed during the upcoming Phase 1 focus groups and interviews with nurses, family caregivers, and care recipients. Summary notes will be taken during all Expert Council meetings.

Next, 3 to 6 focus groups will be conducted in English and French with nurses (1 to 2 groups), family caregivers (1 to 2 groups), and care recipients (1 to 2 groups). Each participant will complete a web-based Qualtrics demographic survey prior to the meetings. Using semistructured interview guides, the interviewer will ask participants about their experiences and interests in respite care, their thoughts on mHealth apps to potentially support palliative-stage family caregiving, and any service coordination apps they currently like or dislike and why. Examples of the key questions and instructions for participants in each phase are listed in Multimedia Appendix 3. The whiteboard, chat, and other key features of the videoconferencing software will be used to help illustrate key points arising from the discussion and promote online engagement. Following these initial discussions, the interviewer will share various potential respite care scenarios that palliative-stage oncology families may find themselves in. Participants will discuss if and how mHealth apps might be used to support the families in those situations.

Finally, follow-up semistructured individual interviews will be conducted with a total of any 8 to 10 participants who agree to be individually interviewed, to gain a more in-depth understanding of participants’ perspectives on mHealth, apps, and respite care [46]. These individual interview participants will be recruited from among participants who participated in the focus groups, or selected from eligible recruits who preferred to only participate in individual interviews.

#### Phase 2: Build and Evaluate Several Low-Fidelity Wireframes

##### Design Cycle 1a: Build Several Wireframes

The Expert Council will review the potential design features identified through the data collected and analyzed in Phase 1. This second Expert Council meeting will focus on achieving consensus as to which design features should be prioritized for the app design. A list of design feature requirements derived from the ongoing data collection, and the creation of a value versus feasibility matrix, will help guide these discussions [47]. Potential design features will be categorized by Expert Council members as being perceived to be (1) of high or low value to the end users and (2) of high or low feasibility to implement in practice. Features that are deemed completely unfeasible to implement and are perceived to be of very low value to end users will be excluded at this stage. All other features will be included, if these features do not prevent the inclusion of the highest priority features (ie, high value, high feasibility).

Using Figma rapid prototyping software [48], the research team will construct several wireframes (ie, low-fidelity/nonclickable proofs of concept) of potential app designs. These wireframes will be based on the Phase 1 rigor and relevance cycle data collected, as well as the Expert Council discussions prioritizing different design features. Creating different wireframes will help prevent premature anchoring of the final design, allowing for more diverse ideas to emerge in subsequent focus groups and interviews [23].

At this time, a member of the research team will begin programming the back-end software needed to make the proofs of concept into a functional app prototype. This software programming will be updated to incorporate new design features identified throughout data collection.

##### Design Cycle 1b: Evaluate Several Wireframes

Next, 3 to 6 semistructured focus groups will be conducted using interview guides designed for Phase 2. At each focus group, the interviewer will screenshare the low-fidelity Figma wireframes of each dashboard. All focus groups will review the wireframes of the family caregiver dashboard. The nurse focus groups will also review the wireframes of the nurse dashboards. Participants will be asked to share detailed feedback on the different proof-of-concept wireframe design features and their perceptions of the potential usefulness of the wireframes. Participants will be asked which of the low-fidelity wireframe features should be prioritized for a future app prototype.

Semistructured individual interviews will also be conducted with any 8 to 10 participants who agree to participate, to gain a deeper understanding of their perceptions of the wireframes. These individual interview participants will be recruited from among participants who participated in the focus groups or from eligible recruits who preferred to only participate in individual interviews.

#### Phase 3: Build and Evaluate an Interactive Proof of Concept of the App and Develop a Functional App Prototype

##### Design Cycle 2a: Build an Interactive Proof of Concept for Usability Testing

The Expert Council will have a third meeting to discuss the ongoing data analyses and the preferred prototype features of the Phase 2 participants. Figma will be used to construct a high-fidelity interactive (“clickable”) proof of concept [48] based on the prioritized design features from the Expert Council meeting. The interactive proof of concept will be combined with Maze usability testing software [49] to create a URL to be shared with participants for online usability testing.

##### Design Cycle 2b: Evaluate the Usability of the Proof of Concept and Program the Final Prototype

Design cycle 2b will be used to quantitatively assess the usability of the high-fidelity, interactive proof of concept in individual test sessions using the Maze usability testing link with the two primary end-user groups: family caregivers and nurses. All new participants will be asked to fill out the Qualtrics demographic survey in advance prior to the meeting, after providing e-consent. Participants will be asked to share their screens, so their assessments of the proof of concept will be video-recorded by the videoconferencing software. Family caregiver participants will be asked to assess the family caregiver dashboard, and nurse participants will be asked to assess both dashboards.

The Maze software will collect usability metrics for effectiveness and efficiency of the proof of concept. Effectiveness will be assessed based on (1) success rate (ie, the proportion of participants who successfully click through the proof-of-concept tasks); (2) the type of errors made by participants while navigating the different features of the proof of concept; and (3) the number of errors made by participants while navigating the proof of concept [49,50]. Efficiency will be measured based on (1) the time spent on specific steps while using the proof-of-concept dashboards and (2) the total time taken for participants to use the proof of concept [49,50].

These data will be analyzed to further refine the interactive proof of concept using Figma software. *Refinement #1* will occur after 7 nurse test sessions and 7 family caregiver test sessions have been conducted. *Refinement #2* will occur after the final 8 nurse test sessions and 8 family caregiver test sessions have been conducted. The new recruits for Phase 3 will be purposively distributed to participate either before Refinement #1 or before Refinement #2, to achieve a roughly equal mix of new perspectives (ie, new recruits for Phase 3) and old perspectives (ie, participants from Phases 1 and/or 2) during Phase 3 data collection. Participants will also be distributed to achieve a roughly equal mix of perspectives from participants with varying levels of comfort with technology based on participants’ demographic questionnaire responses.

The fourth Expert Council meeting will (1) review the findings from Phase 3 and (2) determine the final design features to prioritize for building into the functional app prototype being programmed in parallel, based on general consensus within the Expert Council.

During *Refinement #3,* the interactive proof-of-concept design will be refined based on the Expert Council meeting decisions, and these features will be programmed into the functional app prototype.

### Data Analyses

Data collection and analyses will occur simultaneously, with ongoing discussion with members of the research team. Qualitative data sources will include focus group and individual interview transcriptions; observations and field notes taken during all Expert Council meetings and participant interviews and focus groups; rigor cycle literature review findings; and screenshots of the proof of concept. These data sources will be copied into Excel (Microsoft Corporation) for qualitative content analysis to determine key design features for the app prototype [23,51]. Quantitative demographic survey data will be analyzed using descriptive statistics and displayed in a demographic data table to offer a rich presentation of the characteristics of the participants who informed the app design. Descriptive statistics will also be used to analyze the Maze usability data for the interactive proof of concept. These data will help the Expert Council decide if more data need to be collected to improve the proof of concept prior to the final programming of the functional app prototype.

### Ethical Considerations

The ethical review of this study is pending (McGill University Health Centre, MP-37-2022-7986). There is minimal personal risk involved in participating in this study. In the event that family caregivers or care recipients become distressed, the note-taking member of the research team will ask the participant via a private chat box message if they would like to take a break from the meeting [34]. This research team member will also suggest that the participant follow up with their primary treating clinician at the study site [34]. We will have a list of available resources on-hand for cancer support recommended by the study sites.

## Results

The estimated milestones include (1) 4 months for study setup (eg, ethical approval, hiring and training of personnel, and establishing of the Expert Council key informants); (2) 3 months for Phase 1 recruitment, data collection and analysis; (3) 3 months for Phase 2 recruitment, data collection, and analysis; (4) 3 months for Phase 3 recruitment, data collection, and analysis; and (5) 2 months for final programming of a functional app prototype and knowledge translation. We anticipate that preliminary results will be available by Spring 2022.

## Discussion

We are proposing a new solution to eventually address a significant gap in access to care, namely, access to trusted and flexible respite care services, to ameliorate the current fragmented services rendered to families coping with palliative-stage cancer. To our knowledge, this is the first app being designed to coordinate nurse-provided respite care services to families coping with palliative-stage cancer. A few scholars [21] and industry leaders [52,53] are designing apps for coordinating other forms of respite care services, such as services staffed by nonclinician providers for families coping with age-related chronic health conditions. However, based on our ongoing literature and app store searches, an app for coordinating nurse-provided respite care services, designed with and for palliative oncology families, has not been developed to date.

The proposed research is clinically important because palliative-oncology families require uniquely intensive and skilled respite care services to allow their dying loved ones to remain at home [7,15,41]. Respite care providers without nursing or palliative care training likely do not have these skills, limiting their ability to meet the respite care needs of families coping with palliative-stage cancer [7,16]. Without trusted, flexible, and accessible respite care services, achieving death at home can become an impossible endeavor [5,15]. However, an app for improving the coordination of respite care could have features that would make this endeavor possible. Such features could include flexible scheduling options and choosing among diverse skill sets by the trusted nurse providers of care. These mHealth capabilities could improve the support services rendered to families wishing to support death at home, thus improving the quality of life of patients and their families.

The proposed research is also methodologically important because our rigorous user-centered design study will help to ensure the sustainability of the proposed app-based respite care service by focusing on the needs of end users [22-25]. This app will be collaboratively developed with our transdisciplinary research team of nurse scholars, computer scientists, institutional and community partners, and key informants. With a functional app prototype designed with end users, additional grant applications will be submitted to support future pilot testing and to assess further relevance of the prototype in the field [26]. Although the initial findings will be contextualized to Québec, this innovative methodological approach may be transferable to other populations and settings. Future research could explore the potential of this respite care app to support families with other complex health conditions in other provinces, leading to improved coordination of respite care services across Canada—services that are centered on families’ individualized respite care needs.

## Appendix

Multimedia Appendix 1 Documents confirming the grant support and peer review process by the Rossy Cancer Network Cancer Care Quality and Innovation Research Fund (2020).

Multimedia Appendix 2 Research activities and participant numbers for each phase, displayed in tabular form.

Multimedia Appendix 3 Key questions and instructions for participants during each research phase.

## Abbreviations

ARCC: Canadian Centre for Applied Research in Cancer Control
mHealth: mobile health
