# Supplementary material for: Developing an mHealth Application to Coordinate Nurse-Provided Respite Care Services for Families Coping With Palliative-Stage Cancer: Protocol for a User-Centered Design Study
Source: JMIR Res Protoc. 2021 Dec 13;10(12):e34652. doi: 10.2196/34652 (PMC8713105; doi:10.2196/34652)
Supplement: Multimedia Appendix 2 [file resprot_v10i12e34652_app2.pdf]

Multimedia Appendix 2

The research activities and participant numbers for each phase are displayed below in tabular form.

Phase	Cycle	Research Activities	Participants
**Phases 1-3:**<br>**Literature and app store reviews**	**Rigor cycle 1**	-Academic literature reviews<br>-Google Scholar and Google Search Engine alerts<br>-App store searches	-Not applicable
**Phase 1:**<br><br>**Brainstorm mHealth solutions to respite care scenarios**	**Relevance cycle 1: Brainstorming**	**-Expert Council meeting #1**	-5 key informants and research team
		**-Focus groups (n=3-6),** separate groups for each participant type (nurse, family caregiver, or care recipient) and language (English or French)	***-Nurse focus group(s):***<br>10 nurses in total<br><br>***-Family caregiver focus group(s):***<br>10 family caregivers in total<br><br>***-Care recipient focus group(s):***<br>10 family caregivers in total
		**-Individual interviews (n=8-10)**	-8-10 participants in total from any of the focus groups who agree to participate, or any participants who prefer to solely participate in individual interviews
**Phase 2:**<br><br>**Build and evaluate several low-fidelity wireframes**	**Design cycle 1a: Building**	**-Expert Council meeting #2**	-The same 5 key informants and research team
		**-Rapid prototyping** of several low-fidelity wireframes	-Not applicable
	**Design cycle 1b: Evaluating**	**-Focus groups (n=3-6),** separate groups for each participant type (nurse, family caregiver, or care recipient) and language (English or French)	***-Nurse focus group(s):***<br>10 nurses, either from Phase 1 or new recruits<br><br>***-Family caregiver focus group(s):***<br>10 family caregivers, either from Phase 1 or new recruits<br><br>***-Care recipient focus group(s):***<br>10 care recipients, either from Phase 1 or new recruits
		**-Individual interviews (n=8-10)**	-8-10 participants in total from any of the focus groups who agree to participate, or any participants who prefer to solely participate in individual interviews

**<u>Phase 3:</u>**<br><br>**Build and evaluate an <u>interactive proof of concept,</u> to be programmed into <u>a functional app prototype</u>**	**Design cycle 2a: Building**	**-Expert Council meeting #3**	-The same 5 key informants and research team
		**-Rapid prototyping** of a high-fidelity interactive app proof-of-concept	-Not applicable
	**Design cycle 2b: Evaluating**	**-Individual usability test sessions** of the high-fidelity interactive proof-of-concept (n=14 of the total 30 nurse and family caregiver participants for usability testing)	***Individual test sessions:***<br>-7 nurses, either from the previous phases or new recruits<br><br>-7 family caregivers, either from the previous phases or new recruits
		**-Refinement** #1 of the interactive proof-of-concept	-Not applicable
		**-Individual usability test sessions** of the high-fidelity interactive proof-of-concept (n=16 of the total 30 participants for usability testing)	***Individual test sessions:***<br>-8 nurses, either from the previous phases or new recruits<br><br>-8 family caregivers, either from the previous phases or new recruits
		**-Refinement #2** of the interactive app proof-of-concept	-Not applicable
		**-Expert Council meeting #4**	-The same 5 key informants and research team
		**-Refinement #3** of the interactive proof-of-concept, to be programmed into a functional app prototype	-Not applicable
