# Supplementary material for: Developing an mHealth Application to Coordinate Nurse-Provided Respite Care Services for Families Coping With Palliative-Stage Cancer: Protocol for a User-Centered Design Study
Source: JMIR Res Protoc. 2021 Dec 13;10(12):e34652. doi: 10.2196/34652 (PMC8713105; doi:10.2196/34652)
Supplement: Multimedia Appendix 3 [file resprot_v10i12e34652_app3.pdf]

Multimedia Appendix 3

Key questions and instructions for participants during each research phase are listed below.

<u>Phase 1: Brainstorming ways that a smartphone app could improve respite care services for families coping with palliative-stage cancer</u>

Questions for family caregivers and adults living with cancer:

1) What are your thoughts on using in-home respite care services?
2) For families using respite care services, think back over your respite care experiences. What went well? What didn't go well?
3) For those of you who don't use respite care services, what homecare support services (if any) do you use instead?
4) We know from other studies that families with cancer often experience challenges trusting respite care agencies to provide appropriate care. What are your thoughts on the trustworthiness of respite care services available to families?
5) What are your thoughts on the flexibility of scheduling or coordinating respite care services for families?

Questions for nurses:

1) As nurses, what are your perspectives on in-home respite care for families coping with palliative stage cancer?
2) What are your thoughts on the trustworthiness of respite care services available to the families you care for?
3) What are your thoughts on scheduling or coordinating respite care services for the families you work with?

Questions for everyone: Using apps for coordination or scheduling different services:

1) What aspects of an app make you more comfortable using it? Which apps do you use most frequently?
2) What are your thoughts on using apps for scheduling or coordinating different services? What are your thoughts on using apps like DoorDash for food delivery, Uber for transportation, etc.?
3) What do you think about using apps for scheduling or coordinating different healthcare services?

For each respite care scenario video designed during Phase 1 with the Expert Council, we will ask participants:

1) How could an app be designed to improve this situation, especially for improving the delivery of respite care services?

Questions for everyone: Constructive feedback:

1) Please be our "devil's advocate": what do you foresee going wrong with an app for coordinating nurse-provided respite care?

2) What do you think is the most important thing for us to consider when designing an app-based respite care support service for families, staffed by nurses?
3) Is there anything else regarding in-home respite care, palliative care, and apps that you would like to discuss?

Phase 2: Discussing the features of several possible wireframe designs for an app to improve respite care services for families coping with palliative-stage cancer

Prior to the focus groups, there will be approximately 2-4 different low-fidelity wireframes designed for an app to coordinate respite care services by nurses to families coping with palliative-stage cancer. Family caregiver and care recipient focus groups will only review the family caregiver dashboard of each wireframe design. Nurse focus groups will review both the family caregiver and nurse dashboards of each wireframe design.

For each wireframe:

1) What do you like about the content of this design?
2) What do you dislike about the content of this design?
3) What app features would improve trust in the respite care service?
4) What app features would help create easier access to respite care services?

General questions:

1) Which of these designs would you most want to have turned into an actual app-based service?
2) Which app features absolutely must be in the final app design, for you to ultimately use the app?
3) Which features must not be in the final app design, for you to ultimately use the app?
4) What aspects of the service itself would make you more likely to use it?
5) Is there anything else regarding in-home respite care, palliative care, and apps that you would like to discuss?

Phase 3: Individual testing session guide for nurses and family caregivers

***Family caregiver** participants will only test the family caregiver dashboard of the interactive app prototype. **Nurse** participants will first test their ability to click through the family caregiver dashboard, as if they were a family caregiver requesting respite care. Then, nurses will test their ability to click through the nurse dashboard.*

1) In the chat box, I've shared the link to the app design. Please click on it and let me know when it opens in your browser.

[Request screen sharing.]

2) Now, can you please begin sharing your computer screen with me, so that I can see the app design, too?

3) Please follow the instructions in the link to test out this design.
[Participant clicks through the interactive prototype on their screen. Usability data is recorded on success rate, types of errors while using the app, number of errors while using the prototype (compared to the primary click pathway established by the research team), and time taken to use the prototype.]
