# Supplementary material for: Developing an mHealth Application to Coordinate Nurse-Provided Respite Care Services for Families Coping With Palliative-Stage Cancer: Protocol for a User-Centered Design Study
Source: JMIR Res Protoc. 2021 Dec 13;10(12):e34652. doi: 10.2196/34652 (PMC8713105; doi:10.2196/34652)
Supplement: Multimedia Appendix 1 [file resprot_v10i12e34652_app1.pdf]

Multimedia Appendix 1

The following documents confirm the grant support and peer review process by the Rossy Cancer Network Care, Quality, and Innovation Research Fund (2020).

Réseau de cancérologie Rossy Rossy Cancer Network

1980 Sherbrooke Ouest, #1101
Montréal (Québec)
H3H 1E8

CANCER CARE QUALITY & INNOVATION PROGRAM
RESEARCH FUND 2020 (RF-2020)
LETTER OF AGREEMENT

Dear Dr Argerie Tsimicalis,

The Rossy Cancer Network ("RCN") has awarded a Cancer Care Quality & Innovation Program ("CQI") Research Fund grant to **Dr Argerie Tsimicalis** (the "Principal Investigator" or "PI") for the project, *Developing an mHealth Application to Coordinate Nurse-Provided Respite Care Services For Families Coping with Palliative-Stage Cancer*. The **total amount awarded is $95,147 Canadian dollars**; the Letter of Agreement (the "Agreement") sets forth the terms and conditions of the grant award. Please note that we have deducted $4,805 from your proposed budget as the RCN will not provide funding for article processing fees.

Effective Date and Availability of Research Grant Funds
This Agreement shall be effective as of the signature date ("Effective Date"). Disbursement of funds shall be as follows and subject to PI's compliance with all terms and conditions set forth in this Agreement:

The total amount of the research grant awarded is **$95,147 Canadian dollars**. The funds will be allocated to the PI in **two (2) installments**:

1. **50%** of total award upon receipt of the signed Agreement and **first invoice**, for a total of **$47,573.50 Canadian dollars,** and
2. **50%** of total award (balance) upon RCN written notice of agreement of the mid-year progress report (see "Reporting" section) and the receipt of the **second and final invoice**, for a total of **$47,573.50 Canadian dollars.**

The **term of the Research Project** is **one (1) calendar year.** The **date of the first installment will be considered as the "Start Date"** (T0) of the twelve (12)-month period to complete the research project.

PI must accept the terms of this Agreement before the disbursement of funds. The RCN will manage the transfer of funds to the official financial representative of the host institution, based on the parameters put forth by the RCN gift agreement. Funding must be intra-institution, and an invoice for each payment installment must be provided to the RCN. It is the responsibility of the official financial representative to disburse the funds to the PI. The PI cannot modify or redirect this process.

The billing address on the invoice shall be:
Rossy Cancer Network – McGill University
1980, Sherbrooke Ouest, # 1101
Montréal (Quebec) H3H 1E8

McGill | Centre universitaire de santé McGill McGill University Health Centre | Hôpital général juif Jewish General Hospital | Centre hospitalier de St. Mary St. Mary's Hospital Center

1980 Sherbrooke Ouest, #1101
Montréal (Québec)
H3H 1E8

Communications

The Chair of the Gerald Bronfman Department of Oncology – McGill University will serve as the designate of the RCN Clinical Lead, and act as the official representative of the Cancer Care Quality & Innovation Program and the Program's research and education funding streams.

The RCN will communicate only with the PI for any matter related to the attribution and use of the research grant. Reciprocally, all communications with the RCN representative will be performed exclusively by the PI.

An interim check-in by phone, email, videoconferencing or in-person may be requested at any time during the grant period by either the RCN or PI.

The RCN reserves the right to use any portion of the summary and lay term abstract provided at application for visibility purposes. Also, it may ask the PI for photographs or interviews or any other material deemed necessary for RCN visibility purposes, both within the McGill community and the general public. Communications outside of the PI's immediate team (intra- or inter-institution, outside media, etc.) must be reviewed by the RCN, prior to circulation.

Unused Funds and Extension

After the grant period, PI agrees to return any unexpended or unaccounted for funds to RCN or to submit a written request for an extension of the grant period. PI agrees to return all disbursed funds if (1) grant funds have not been used for their intended purpose, or (2) have been used inconsistently with the terms of this Agreement, or (3) if the activities or outputs funded by the grant are materially incomplete by the end of the grant period, as determined by RCN in its sole discretion.

Original receipts and invoices must be maintained by the PI and shall be made available to RCN upon request. PI agrees to maintain adequate financial records consistent with generally accepted accounting practices. RCN may monitor and evaluate PI's operation to ensure compliance with Agreement.

Intellectual Property

PI owns any intellectual property it produces within the scope of this grant.

Ethics Clearance

The RCN is not responsible for securing ethics clearance for any funded project. This is the exclusive responsibility of the PI, and must be obtained for all clinical sites involved in the project. Confirmation of ethics clearance must be included in the mid-term progress report – scientific narrative section.

1980 Sherbrooke Ouest, #1101
Montréal (Québec)
H3H 1E8

Obligations
PI will notify RCN of any media queries or publications or other materials resulting from the grant no later than ten (10) business days in advance of distribution or publication. PI will acknowledge RCN's support at the beginning of any presented material or publication referring to or resulting from this grant, see *Communications Policy and Guidelines for RCN Funded Projects* attached as Exhibit C.

PI agrees that RCN may include information about the PI and its activities within its own communication channels and social media platforms as well annual reports and may distribute such information to third parties.

Upon request of the RCN, the PI is expected to attend RCN-related scientific and clinical, visibility-related events during the twelve (12) months corresponding to the funding start to present work accomplished.

The PI named on the original proposal submitted cannot be modified for the duration of the grant unless otherwise approved in writing by the RCN. PI may not assign this Agreement or delegate performance of the terms of this Agreement or of the project to any other person or entity without RCN's prior written consent.

The PI is solely responsible for all activities supported by the awarded research grant. Nothing in this Agreement creates a partnership, agency, joint venture, employment, or any other type of relationship. The PI shall not represent itself as an agent of RCN for any purpose and has no authority to bind RCN in any manner whatsoever.

The person(s) signing this Agreement on behalf of PI represents and warrants to RCN that he or she has the requisite legal authority and power to execute this Agreement and to bind PI to the obligations contained herein.

Please return the signed original of this letter to **Dr Gayle A. Shinde**r, keeping a copy for your records.

This Agreement has been drafted in the English language at the express request of the parties.

[signature page follows]

Réseau de cancérologie Rossy
Rossy Cancer Network

1980 Sherbrooke Ouest, #1101
Montréal (Québec)
H3H 1E8

SIGNATURES

On behalf of the **ROSSY CANCER NETWORK** and the **GERALD BRONFMAN DEPARTMENT OF ONCOLOGY – MCGILL UNIVERSITY**:

Signature: ______________________

Name: **Dr Eduardo L. Franco O.C., DrPH, PhD (Hon.), FRSC, FCAHS**
Title: **James McGill Professor and Chair, Gerald Bronfman Department of Oncology**
Director, Division of Cancer Epidemiology, McGill University – Faculty of Medicine

Date: April 13, 2021

Agreed to and accepted on behalf of the **PRINCIPAL INVESTIGATOR**:

Signature: ______________________

Name: **Dr Argerie Tsimicalis**
Title: **Associate Professor, Ingram School of Nursing**

Date: April 14 2021

Encl. Research Fund – 2020 Proposal and supporting documents (Exhibits A and B)
Communications Policy and Guidelines for RCN-funded projects (Exhibit C)

The following Rossy Cancer Network Research Fund reference document describes the fund and peer-review process.

https://www.mcgill.ca/rcr-rcn/funding/research/2020-edition

Réseau de cancérologie Rossy | Rossy Cancer Network

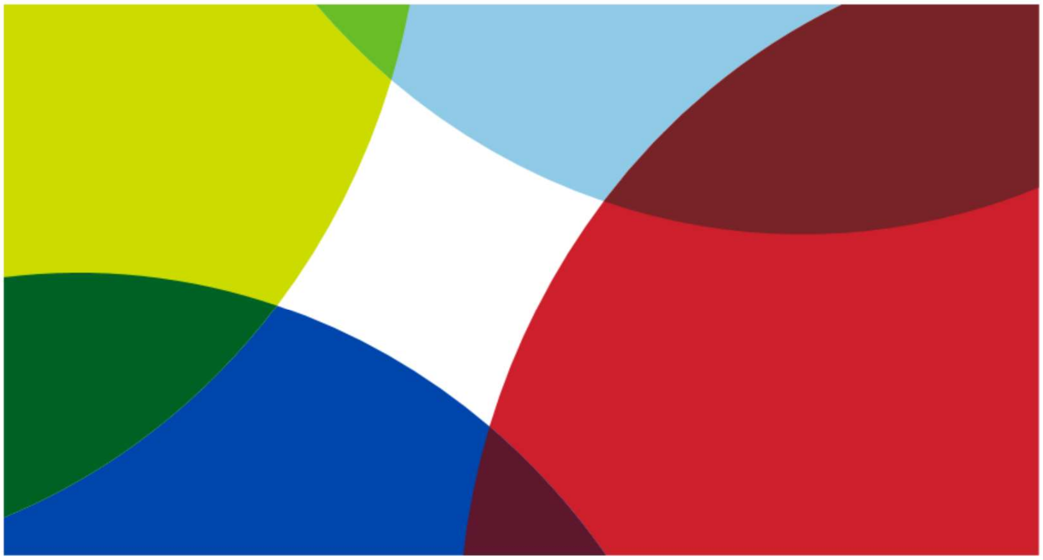

CANCER CARE QUALITY INNOVATION PROGRAM (CQI)

RCN Research Fund

2020 Edition

Reference Document

McGill | Centre universitaire de santé McGill / McGill University Health Centre | Hôpital général juif / Jewish General Hospital | Centre hospitalier de St. Mary / St. Mary's Hospital Center

Summary

The RCN Research Fund was established by the RCN Cancer Care Quality & Innovation program to support advancements in cancer care quality research. This initiative builds on the exceptional pool of expertise across the partner institutions that make up the Rossy Cancer Network (RCN): the McGill University Health Centre, the Jewish General Hospital, St. Mary's Hospital Center, and the McGill University Faculty of Medicine.

This fund helps to support the unprecedented collaboration among the McGill-affiliated cancer missions which is at the heart of the RCN: promoting continuous improvement projects, sharing best practices and developing research and training platforms for the rapid generation of scientific knowledge related to cancer care quality.

The Research Fund is accessible to all health professionals directly or indirectly involved in the provision of cancer care and promotes collaboration among the network's partner hospitals.

Since 2014, the RCN Research Fund has invested over $2 million to enable the completion of 29 projects targeting various aspects of the cancer care trajectory. This exciting initiative has empowered health care teams to continually improve cancer care delivery and enhance patient experience and outcomes across the network, while advancing knowledge in cancer quality and strengthening McGill's leadership in this field.

Purpose

- Support the RCN Cancer Care Quality & Innovation program's mandate of academic advancement in cancer care quality and safety;
- Assist McGill's oncology community in becoming a recognized leader in the field of cancer care quality research and innovation;
- Facilitate the deployment and execution of RCN strategic priorities and vision.
- Assist McGill's oncology community in ensuring regional leadership in establishing high standards in cancer care quality and clinical effectiveness; and optimizing the patient trajectory (diagnosis, treatment planning, treatment delivery, survivorship, supportive care and end of life);
- Develop research and training platforms for the rapid generation of scientific knowledge relevant to the domains of cancer care;
- Facilitate the academic career development of faculty members within the McGill oncology community involved in research in the field of cancer care quality.

Strategic Pillars

The RCN Research Fund will only support proposals that are aligned with one of the following 5 priority pillars:

1. **<u>Access to Specialized Care</u>**:
   The RCN will develop & implement a mechanism to allow patients to have timely access to the network, no matter where their initial point of entry is.

2. **<u>Harmonized Tumor Boards</u>**:
   The care that is prescribed and the standard of care that a patient would receive should be at the same high level, based on evidence and tailored to the patient, regardless of what tumor board discussed their medical case.

3. **<u>Access to clinical trials:</u>**
   Expanded access to clinical trials across all the RCN facilities improves the quality of care by allowing access to novel treatments, better disease management, and enhanced prognostic surveillance.

4. **<u>Trajectories of Care</u>**:
   Improved case management assures continuity of care for all the patients' health needs via inter-team and inter-hospital coordination with seamless integration of clinical data among RCN's institutions.

5. **<u>Proximity of treatment of care:</u>**
   Whenever possible, the best cancer treatment and care beginning with early diagnosis through to recovery and/or palliative care, as well as perioperative care, should be delivered primarily close to the patient's home.

Eligibility

RCN Research Grants are open to ***all health care professionals*** actively involved in the delivery of care to cancer patients or ***cancer researchers*** affiliated with McGill University whose research program can have clear and direct benefits for the delivery of cancer care within the RCN network of hospitals. They are available ***to all*** with an interest in the development of new knowledge and advancement in cancer care optimization. The list of eligible professionals includes, but is not limited to:

- Physicians
- Nurses and all nursing-affiliated personnel
- Rehabilitation experts (physiotherapy, occupational therapy, kinesiology, speech therapy, etc.)
- Psychosocial health experts
- Epidemiologists and health economists
- Other professionals that participate in the delivery of care to cancer patients

The budget proposal for each application must not exceed $100,000. All applications must be submitted online at https://www.mcgill.ca/rcr-rcn/grants/cqi-research/submit by Friday January 15, 2021 (The complete submission must be received **no later than midnight** on January 15, 2021). Applications submitted by email in whole or in part, will not be considered.

Note: The RCN Executive Committee has approved the following measures to minimize the potential for conflict of interest: 1) The RCN Clinical Lead/hospital Oncology Lead (Dr Armen Aprikian) is not eligible for funding. 2) The other hospital Oncology Leads (Drs Gerald Batist & Adrian Langleben) are eligible to participate as co-applicants only. They will not be involved in ranking applications. 3) The Chair of the Department of Oncology (currently Dr Franco) is not eligible to compete for funding.

Application Terms and Conditions

- There is no limit on the number of research proposals submitted by one individual (as a principal investigator, co-applicant or collaborator); however the same individual can only receive one research award as a principal applicant per year.

- All applicants and co-applicants must hold a professional or clinical appointment in at least one of the RCN-partner institutions: The McGill University Health Centre (MUHC), the Jewish General Hospital (JGH) and St-Mary's Hospital (SMH). External collaborations can be sought; however, budget expenditures to non-RCN-partner institutions require specific justification and approval. McGill-affiliated faculty members are also eligible.

- Research proposals must reflect a collaborative effort from ***at least 2 RCN-partner institutions.*** The contribution of each collaborating partner must be proportionally equivalent (development and deployment of the research project, data analysis and interpretation) and cannot be limited to patient accrual.

- Research proposals must contain a knowledge transfer component (there are no restrictions on the type, level and professional affiliation of the targeted audience).

- Only original proposals will be considered. The RCN will not award funds if overlapping or comparable support for the same project has been obtained (even partially) from another funding source. Should the proposal subsequently get funding from another source, the corresponding amount will be withheld.

- Research proposals having received funding through previous RCN funding cycles are not eligible for funding. This also applies to new proposals that represent the continuation of projects funded through previous cycles of RCN funding (Research Fund or Quality Improvement Initiatives program).

- The principal investigator is responsible for producing all relevant ethics approvals from his/her institution and will provide documentation of such to the RCN within 6 months of proposal approval.

- Matching contributions from host Departments or Institutions (e.g. in-kind resources, funds, space, equipment, dedicated personnel, etc.) are not a mandatory requirement.

- Proposals must comply with the application requirements set forth by the RCN Cancer Care Quality & Innovation program (application form, budget proposal, letter of support, etc.). Proposals that fail to do so will be excluded despite potential scientific merit.

- Research funds cannot be used to: (i) support clinical trials, (ii) support travel or meetings, (iii) support indirect expenditures (e.g. leasing or maintenance, indirect costs by host institutions), (iv) supplement or replace institutional / clinical operating budgets, (v) provide salary support for any of the applicants or collaborators.

Review Process

- Applications will be reviewed by a **scientific peer-review committee** that may include previous recipients of the RCN Research Fund. The committee will be chaired by the Chair of the Gerald Bronfman Department of Oncology (or delegate). A standardized scoring grid will be used (provided in this document).
- The role of Chair of the scientific peer-review committee will be to advise the committee members on issues such as scoring criteria, terms and conditions and eligibility of proposals.
- Three committee members will independently score each application, followed by a discussion session to finalize the rank list.
- Scientific merit will be the sole criterion; the RCN does not have any obligation to ensure parity of funding between sites, professionals and teams.
- The scientific committee's rank list will be presented by the Chair of the Gerald Bronfman Department of Oncology (or delegate) along with the recommendations for discussion at the Executive Committee, to ensure that funding decisions are aligned to RCN goals and values.
- The Executive Committee is responsible for determining the RCN Research Fund's annual budget, maximum number of awards distributed and proposals selected.

Reporting Process for Recipients

- Financial and scientific progress reports are required at 6 months and 12 months.
- A 12-month **no-cost** extension can be requested upon submission of the first 6-month progress report. A written official letter and new timeline with action plan is required. Consequently, the RCN leadership acknowledges the carry-over of assigned funds to the following fiscal year, without penalty to the CQI budget. Candidates who have been exceptionally granted a one-year extension would have to keep providing update reports (financial & scientific) until the final deadline has been reached.
- The principal investigator is expected to document planned carryovers (of funds or data collection) and provide an update within 1 year.
- Final financial and scientific final reports are required 6 months (maximum) after the end of a project, for both internal and external communications.
- Principal investigators should use standardized templates for progress and final reports provided by the Gerald Bronfman Department of Oncology.
- Recognizing the unique financial interplay between the Faculty and RCN-partner hospitals which may result in awards being disbursed at variable dates, the day that the 1st installment is made to the host institution will be considered as the start date of the research project and not the day on which notification of award was made to the grant recipient.

Acceptance Terms and Conditions

- **Principal Investigator (PI):** the PI named on the original proposal submitted cannot be modified during the funding period (12-month period or accordingly to extension approved).

- **RCN Research Fund representative:** The RCN Research Fund is managed by the Gerald Bronfman Department of Oncology. The Department Chair and the Chair's Senior Advisor for Academic Programs will act as the official representatives of the RCN Research Fund. However, disbursement of funds will be handled by the Rossy Cancer Network.

- **Correspondence:** the RCN Research Fund representatives and the RCN will communicate only with the PI for any matter related to the attribution and use of the research grant. Reciprocally, all communications with the RCN Research Fund representatives or the RCN shall be performed exclusively by the PI.

- **Ethics clearance**: the RCN is not responsible for securing ethics clearance for any funded project. This is the exclusive responsibility of the principal investigator.

- **Transfer of funds**: the RCN Financial Officer will manage the transfer of funds to the local research institute, based on the parameters of the RCN gift agreement. Funding must be intra-institution, triggered by an invoice from the research institute to RCN. It is the responsibility of the local financial representative to disburse the funds to the PI. The PI cannot modify or redirect this process.

  Once the contract has been signed, research funds are attributed in 2 installments of 50% of the total granted amount. The date of the first installment will be considered as the start date (T0) of the 12-month period to complete the research project. The second installment will be made upon reception and approval of the 6-month progress report.

- Funding is not recurrent. The RCN reserves the right to re-claim any unused funds at the end of the research project.

- **Amount:** the amount specified on the acceptance letter will not be modified under any circumstance. If applicable, the PI is encouraged to leverage this financial support from the RCN to apply for external sources of funding.

- **Reporting requirement (financial**): A financial report is required at 6 months, detailing expenses according to the standard RCN format provided. Expenses not justified in the original proposal will be subject to review and potential rejection. A final financial report is required at 18 months for project completion. The RCN reserves the right to re-claim any unused funds at that time.

- **Reporting requirement (scientific):** the PI is required to produce a scientific report at 6 months, detailing progress of the project, according to the standard RCN format provided. Subsequent payment of funds can be withheld if the progress is judged non-satisfactory, after discussion with the Executive Committee. The final scientific report is required a maximum of 6 months after project completion.

- **Communications:** the RCN reserves the right to use any portion of the summary and lay term abstract provided at application for promotion purposes. In addition, it may ask the PI and his/her team for photographs or interviews or any other material deemed necessary for RCN promotion purposes both within the McGill community and the general public. The grantee also agrees that the RCN will manage media and communications related to the funded proposal. Communications outside of the PI's immediate team (intra- or inter-institution, outside media, etc.) must be reviewed by the RCN communications Manager.

- **Acknowledgement**: The grantee agrees to acknowledge the RCN in all written or oral forms of communications \ stemming from funded proposal. Please refer to the Communications Policy for CQI projects available online at mcgill.ca/rcr-rcn/rcn-initiatives/team-tools

- **RCN-related events**: The PI is expected to attend RCN-related scientific and clinical events during the 24 months corresponding to the funding start to present work accomplished upon request of the RCN.

Additional Information

For questions regarding eligibility, please contact:

Dr Gayle Shinder
Chair's Senior Advisor for Academic Programs
Gerald Bronfman Department of Oncology
Email: cqi.onc@mcgill.ca

Assessment Grid

CRITERIA	SCORE
**1. Quality and originality of proposal:**<br>-The project proposed is original and innovative<br>-The project targets cancer care quality improvement<br>-The project includes an objective assessment component<br>- Clarity of the proposal (problem statement well documented, research questions clearly stated, objectives measurable and tangible outcomes)<br>-The objectives correspond to the hypothesis and are realistically attainable within the timeline and methods proposed	/4
**2. Feasibility of the proposal within proposed teams and environments:**<br>- The methodological approach proposed is appropriate, realistic and feasible according to the research goals (e.g. number of participants, accessibility of participants, ethics considerations etc.)<br>-Technical Capacity : Team composition covers all relevant clinical / scientific skills required<br>-The clinical / scientific activity of the proposed sites is able to support the proposed research activity	/4
**3. Relevance of project to cancer care quality & research:**<br>-The project targets an unanswered question in the science<br>-The project builds on currently existing knowledge<br>-The research question is relevant<br>- The proposed research is likely to have an impact by leading to ground-breaking advances in cancer care quality improvement<br>- Results from the proposed project will support larger research initiatives<br>- Results from the proposed project has the potential to be rapidly translated into practice and/or guidelines	/4
**4. Budget relevance:**<br>-The proposed budget realistically reflects the requirements to successfully complete the project (sufficiency)<br>-The proposed budget covers all aspects to successfully complete the project (comprehensiveness)<br>-The proposed budget does not contain elements corresponding to institutional operating expenses<br>-The proposed budget is in compliance with the terms and conditions<br>-The proposed project could realistically persist beyond research activities with reasonable, minimal support (sustainability)	/4
**5. Relevance to the RCN goals and objectives:**<br>- The proposal is relevant and align with RCN objectives and strategic priorities (Patient Experience, Evidence based-care, Access to care, Leading-Edge treatments, Research and Education)-<br>The project involves more than one site as proportionally equal partners (clinical and scientific collaboration, patients recruitment…etc)<br>-The project will generate knowledge / know-how or opportunities that can be exported to other RCN sites, priorities or initiatives<br>-The project has the potential for eventual alignment with disease site group activities	/4

The following website confirms the 2020 recipients of the research fund competition, with this project listed on page 3:

https://www.mcgill.ca/rcr-rcn/funding/research/recipients

Fr (/rcr-rcn/fr/bourses/fonds-de-recherche-cqi/cqi-boursiers)

Réseau de cancérologie Rossy
Rossy Cancer Network

(/rcr-rcn/)

Search

McGill.CA (https://www.mcgill.ca) / ROSSY CANCER NETWORK (HTTPS://WWW.MCGILL.CA/RCR-RCN) / Funding Opportunities (/rcr-rcn/funding) / Research Fund (/rcr-rcn/funding/research)

2020

Three projects totaling $287,537 were awarded funding in the 2020 edition of the Rossy Cancer Network's CQI Research Fund competition:

Surgery - Rectal Cancer

Early ileostomy closure following restorative proctectomy for rectal cancer patients: A North American multicenter randomized-controlled trial (RCT)

Patients undergoing rectal cancer surgery typically need a temporary stoma ("bag") in order to protect the new connection. While the stoma is meant to prevent consequences of a leak, up to 30% of patients experience complications from the stoma. Patients report that their quality of life is affected negatively while living with a stoma and there is also an associated financial burden. Studies in Europe have shown that some patients can have the stoma safely closed within two weeks of rectal cancer surgery. These patients have fewer complications while they have their stoma and also experience fewer complications following stoma reversal compared to patients who undergo standard of care closure of their stoma. Unfortunately, this practice has not been adopted in North America. We have designed a trial in 7 hospitals in North America to assess early stoma closure and its benefits in North American patients. We will also study its impact on patients' quality of life and cost.

Marylise Boutros will lead a JGH-MUHC-SMHC team including co-investigators, Carol-Ann Vasilevsky, Allison Pang, Té Vuong, Petr Kavan, Gerald Batist, Vincent Pelsser, Huy Le, Anne Ross, Carmen Loiselle, Sender Liberman, Caroline Reinhold, Neil Kopek, Sebastien Demyttenaere, Fadi Habbab and Natasha Caminisky (trainee) from the RCN network as well as external collaborators, Rebecca Auer from The Ottawa Hospital and Sunil Patel from Kingston Health Science Centre.

Patient Experience - Lung Cancer

The use of Patient Report Outcomes (PROs) to improve integrated care among patients diagnosed with lung cancer and treated across different cancer centres

This study aims to improve the care of lung cancer patients across the trajectory of care by asking them about the symptoms they experience (either due to the cancer itself or its treatments), communicating this information back to clinicians, and facilitating follow-up symptom management care. This might have an impact on how well individuals can manage their treatment and on the quality of their day-to-day lives. Patients may also be treated by different care providers, often across different healthcare settings, and this study will explore how to improve communication among providers. To achieve our aims, we will partner with patients and care providers to design the symptom screening program that will be delivered electronically. Through a series of group meetings, patients, care providers, and researchers will work together to identify the priorities and content of this screening program. We will then test the screening program with a small group of patients with lung cancer to make sure it does in fact meet the priorities of all end users.

Sylvie Lambert and Rosanna Faria will co-lead a SMHC-MUHC team including co-investigators, John Kildea, Tarek Hijal, Myriam Ménard, Benjamin Shieh, Jonathan Spicer and Christine Bouchard and collaborators, Adrian Langleben, Sergio Faria and Scott Owen.

Patient/Family Caregiver Experience - Palliative-Stage Cancer

Developing an mHealth Application to Coordinate Nurse-Provided Respite Care Services For Families Coping with Palliative-Stage Cancer

Cancer is a condition requiring ongoing family caregiving support, particularly during the palliative stage of care. Without adequate support services, family caregivers are at risk for depression and poor health, and

loved ones with cancer are at risk for inadequate care. For such families, in-home respite care can be invaluable. Yet, respite care services are under utilized,largely due to the inflexibility of services and families' lack of trust in providers' abilities. For families with complex care needs, nurses may be best positioned to provide trusted and flexible respite care. Mobile applications ("apps") are being used in other industries to make services more flexible. However, no apps have been developed to coordinate nurse-provided respite care services. We aim to develop an app that can optimize the coordination of nurseprovided respite care services for families coping with palliative-stage cancer, thus improving the quality of services rendered to these families.

Argerie Tsimicalis will lead a MUHC-JGH team including co-investigators, Antonia Arnaert, Vasiliki Bitzas, John Kildea and Karyn Moffatt and collaborator, Aimee Castro.

Past years

2019 (//www.mcgill.ca/rcr-rcn/funding/research/recipients/2019)
2018 (//www.mcgill.ca/rcr-rcn/funding/research/recipients/2018)
2017 (//www.mcgill.ca/rcr-rcn/funding/research/recipients/2017)
2016 (//www.mcgill.ca/rcr-rcn/funding/research/recipients/2016)
2015 (//www.mcgill.ca/rcr-rcn/funding/research/recipients/2015)
2014 (//www.mcgill.ca/rcr-rcn/funding/research/recipients/2014)

Accessibility (https://www.mcgill.ca/accessibility)

Log in 🔒 (/rcr-rcn/saml_login?destination=node/1401)
